# Perceived Social Status and Suicidal Ideation in Maltreated Children and Adolescents

**DOI:** 10.1007/s10802-021-00852-7

**Published:** 2021-08-11

**Authors:** Kelli L. Dickerson, Helen M. Milojevich, Jodi A. Quas

**Affiliations:** 1 Department of Psychological Science, University of California Irvine, Irvine, CA USA; 2 Center On Child Abuse and Neglect, University of Oklahoma Health Sciences Center, Oklahoma City, OK USA

**Keywords:** Suicidal ideation, Child maltreatment, Subjective social status, Adolescents, Preadolescents

## Abstract

Recent decades have seen an alarming increase in rates of suicide among young people, including children and adolescents (“youth”). Although child maltreatment constitutes a well-established risk factor for suicidal ideation in youth, few efforts have focused on identifying factors associated with maltreated youths’ increased risk for suicidal ideation, especially across development. The present study examined the relations between maltreated youths' (*N* = 279, *M* = 12.06 years, 52% female, 53% Latinx) perceptions of their social status and suicidal ideation and compared those relations between pre-adolescents and adolescents. Findings revealed unique developmental patterns: Perceived social status was associated with suicidal ideation, but only in adolescents, who showed greater risk for suicidal ideation if they viewed themselves as lower ranked in society and lower risk for suicidal ideation if they viewed themselves as higher ranked in society. Findings have implications for scientific and practical efforts aimed at better understanding and preventing suicide in a high-risk developmental population.

Suicide is a serious global public health concern, especially among young people. Recent decades have seen a rising trend in youth suicide rates, with suicide now constituting the second leading cause of death among individuals ages 10–34 in the United States and among 15–29-year-olds world-wide (Centers for Disease Control and Prevention [CDC], 2018; World Health Organization [WHO], 2014). Between childhood and adolescence, suicidal thoughts and behaviors increase markedly, and the progression from ideation to attempt occurs fairly rapidly for a majority of adolescents (i.e., within a year of onset of ideation; Nock et al., 2013). Because suicidal ideation is one of the most robust predictors of eventual suicidal behavior, understanding such ideation in youth, particularly among high-risk youth, is of paramount importance in developing responsive prevention efforts.

Child maltreatment – another global public health concern, affecting an estimated one billion children in the past year (Hillis et al., 2016) – is a well-known predictor of suicidal ideation. All forms of child abuse (i.e., physical, sexual, emotional) and neglect have been associated with increased suicidal ideation and suicidal behavior in youth (Miller et al., 2013, 2017). Still, not all youth who endure maltreatment go on to develop suicidal ideation. Indeed, recent studies suggest that, despite elevated rates of suicidal ideation among maltreated relative to non-maltreated youth (i.e., rates 3–9 times higher), less than a third (i.e., 7–27%) of maltreated youth actually report such ideation (Anderson, 2011; Cicchetti et al., 2010; Evans et al., 2017; Pilowsky & Wu, 2006)*.*

Given variability in rates of suicidal ideation among maltreated youth, of significant interest has been investigations into which specific characteristics in this population lead some but not others toward risk for suicidal ideation (for a review, see Miller et al., 2013). One such characteristic is female gender (Anteghini et al., 2001; Baldry & Winkel, 2003; Miller et al., 2017), a characteristic that has consistently emerged in other populations: Girls in general are at greater risk for suicidal ideation and self-harm than are boys (Griffin et al., 2018; Miranda-Mendizabal et al., 2019; Nock et al., 2013). Other characteristics, though, are more specific to youths’ maltreatment experiences. For instance, experiences of physical, sexual, and/or emotional abuse are more strongly and consistently associated with suicidal ideation than are experiences of neglect only; as are greater severity and chronicity of abuse (Dube et al., 2001; Finzi et al., 2001; Johnson et al., 2002; Thompson et al., 2005; Thompson et al., 2012). Finally, inter- and intra-personal characteristics in maltreated populations, such as specific genetic polymorphisms (i.e., 5-HTTPLR short or short/long genotypes), low levels of perceived social support, poor problem-solving abilities, and substance use, have also been associated with enhanced risk for suicidal ideation in maltreated youth (Cicchetti et al., 2010; Esposito & Clum, 2002; Miller & Esposito-Smythers, 2013).

Beyond the aforementioned experiential characteristics, how maltreated youth *perceive* themselves and their lives may also confer risk for suicidal ideation. Interest in perceptions stems in part from theoretical models, including the cognitive-behavioral theory of adolescent suicide and the cognitive-vulnerability hypothesis, which contend that significantly stressful or traumatic life events may trigger negative perceptions that place adolescents at heightened risk for suicidal ideation, and ultimately, suicidal behavior (Alloy et al., 2000; Spirito et al., 2011). Equally important, though, is practical interest, given that perceptions, unlike past experiences, may be amenable to change in order to reduce risk. Some empirical research, including with maltreated populations, is consistent with theoretical models: Maltreatment is associated with global negative views of oneself, the world, and future, and such views have been further linked with risk for suicidal ideation in maltreated populations (Miller & Esposito-Smythers, 2013; Miller et al., 2016; Ponce et al., 2004). Other work, however, suggests more nuanced perceptions may also be relevant to suicidal ideation in youth, especially youths’ perceptions of their place in the social hierarchy, referred to as subjective social status (Ko et al., 2014; Wetherall et al., 2019).

For instance, several studies suggest that suicidal ideation and attempts are significantly more common among adolescents and young adults who perceive themselves to be of lower social status than their peers than among adolescents and young adults who see themselves as higher in social status (Goodman et al., 2017; Jeon et al., 2013; Ko et al., 2014). Similar associations between such perceptions and other mental health problems often linked to suicidal ideation have also been reported. For instance, adolescents who report low subjective social status evidence increased depressive symptoms, anxiety, attention problems, substance use, conduct problems, and crime during adolescence and young adulthood (Goodman et al., 2015; McLaughlin et al., 2012; Quon & McGrath, 2014; Rivenbark et al., 2019, 2020; Russell & Odgers, 2020), and show greater psychological and physiological responses to stress (e.g., greater fear reactivity, faster cortisol reactivity; Rahal et al., 2020). Of importance, the associations between perceived social status and functioning remain robust even after controlling for objective indicators of socio-economic status (e.g., family income, education, resources), suggesting that perceptions are uniquely influential in predicting risk for mental health problems, including suicidality (e.g., Rivenbark et al., 2019, 2020).

When considering maltreated youth specifically, it is likely that the links between perceived social status and suicidal ideation both exist and are particularly robust. First, maltreatment, especially abuse (i.e., sexual, physical, and psychological maltreatment), contributes to a range of negative or distorted self-perceptions, including a sense of isolation, unwantedness, low self-esteem, and shame, all of which may reduce youths’ feelings of belonginess and value within society (Bennett et al., 2005; Calheiros et al., 2020; Elliott et al., 2005; Kim & Cicchetti, 2006; Sekowski et al., 2020). The consequences of abuse, which often include interpersonal problems (e.g., hostile attribution bias, aggression), mental health symptoms (e.g., depression, anxiety), and for some, removal from home and placement into foster care, may reinforce youths' negative self-perceptions (Cicchetti & Toth, 2005; Harms et al., 2019). In addition, many of the objectively adverse experiences that often accompany maltreatment may further contribute to these youths’ low perceptions of their societal value. Maltreated youth often come from economically disadvantaged backgrounds and reside in communities characterized by violence, concentrated poverty, and weak social connections (Freisthler et al., 2006; Maguire-Jack & Font, 2017; Storer et al., 2014), which, when combined with the maltreatment itself and its consequences, may well lead to particularly low perceptions among maltreated youth about their societal value, especially relative to others. These perceptions, in turn, place them at significant risk for suicidal ideation and behaviors.

Finally, while little attention has been paid toward possible developmental changes in the links between subjective social status and suicidal ideation, including in maltreated youth, development is nonetheless an important consideration. For one, with the transition from childhood to adolescence, youth become increasingly sensitive to social comparisons and evaluations (Crone & Dahl, 2012) and begin to develop a more socially-integrated sense of self (Sebastian et al., 2008). These changes, plus cognitive advances with development (e.g., in the ability to think abstractly), likely afford adolescents a more sophisticated understanding of their social position (Goodman et al., 2001; Steinberg & Morris, 2001). Second, adolescence marks the onset or exacerbation of mental health symptoms, including suicidal ideation, for many youth, especially girls and those who have endured maltreatment (Adrian et al., 2016; Kessler et al., 2005; Miller et al., 2013; Nock et al., 2013). Theoretically, changes in how adolescents perceive their social position and the increasing salience of social status concerns during adolescence may lead to stronger associations between status-related perceptions and suicidal ideation in adolescence (Steinberg & Morris, 2001). Both population-representative and longitudinal cohort studies of adolescents suggest that, with age, subjective social status becomes more accurately calibrated to objective indices of social position (e.g., household SES) and more strongly associated with mental health and well-being (e.g., depression, anxiety, conduct problems; Goodman et al., 2015; Rivenbark et al., 2020). Thus, the links between status-related perceptions and suicidal ideation may well become stronger with development.

Despite this possibility, a key limitation of extant findings concerns their predominant focus on older adolescents. Even preadolescents’ (i.e., ages 10–13) perceptions of social status track objective (i.e., family, school, and neighborhood-level) indicators of socio-economic status, suggesting that such youth already have a general understanding of their socio-economic reality (Rivenbark et al., 2019). Younger children, as well, recognize and can report on their status in social hierarchies (Thomas et al., 2018; Thomsen, 2020), although whether they can do so with regard to socio-economic hierarchies and whether differences in their perceptions relate to suicidal ideation is not known, especially among maltreated youth, who are at heightened risk for such ideation.

Overall, insofar as perceptions of social status are related to suicidal ideation in maltreated youth, such perceptions may represent a relatively simple and modifiable target for efforts aimed at preventing suicidal thoughts and behavior in maltreated youth. At the same time, though, it is imperative to assess whether such relations change as youth transition into adolescence so that intervention efforts can be targeted toward those developmental windows when such perceptions may exert their strongest effects.

## Present Study

In the current investigation, we evaluated subjective social status and suicidal ideation in youth, aged 6–17 years, who had been removed from home due to substantiated maltreatment.^1^ During their stay at a temporary residential facility, the youth completed questionnaires assessing their perceived social standing, mental health symptoms, and suicidal ideation. Information regarding their maltreatment history was obtained via social service records and compared to their subjective social status and endorsement of suicidal ideation. Given the potential greater salience of abuse versus neglect to suicidal ideation and self-perceptions (e.g., Bennett et al., 2005; Miller et al., 2013), we were particularly interested in potential differences in associations between youth who suffered abuse versus those who suffered primarily neglect. Based on extant literature, we expected that suicidal ideation would be significantly more common among youth who had histories of abuse exposure relative to those with exposure to neglect only, and inversely related to youths’ subjective social status. We also expected that the links between subjective social status and suicidal ideation would be more pronounced in adolescents than in preadolescents. Finally, we predicted that differences in subjective social status would moderate the associations between abuse and suicidal ideation in youth.

## Method

### Participants

The sample included 279 children and adolescents ages 6–17 (*M* = 12.06, *SD* = 3.22; 52% identified as female, 48% as male) recruited from a temporary residential facility on the west coast of the United States. All youth had experienced maltreatment that was both substantiated by social services and considered sufficiently serious to warrant their removal from parental custody. Youth were divided into two age groups, following developmental cut-offs reflecting whether they were preadolescents (ages 13 and younger; *n* = 176) or adolescents (ages 14 and older;* n* = 103) (for similar approaches, see Cicchetti et al., 2010; Rivenbark et al., 2019). We divided youth in this way both to be consistent with prior work on subjective social status and mental health and given evidence that these perceptions might operate differently across the preadolescent versus adolescent period (e.g., Goodman et al., 2001; Rivenbark et al., 2019). A majority of the sample identified as Latinx (53%), followed by multi-ethnic (17%), other (17%), and White (13%), and a majority of youth came from low socioeconomic backgrounds, determined via geographic coding of the neighborhoods where youth had lived prior to removal. According to social services records, 39% of youth had experienced physical abuse, 19% had experienced sexual abuse, and all youth had been exposed to some form of neglect (i.e., lack of supervision or failure to protect; information relevant to emotional maltreatment was largely lacking and thus not considered further). In exactly half of cases, more than one form of maltreatment was documented, with 41% of youth being exposed to two forms of maltreatment and 9% exposed to all three (see Procedure for maltreatment coding and reliability). Given the significant overlap among maltreatment subtypes and the high rates of neglect relative to other forms of maltreatment, for analytic purposes, we heuristically grouped youth into two categories reflecting abuse exposure (substantiated physical and/or sexual abuse, regardless of neglect exposure; *n* = 139) versus substantiated neglect only (*n* = 140).

We also collected information about youths’ experiences once maltreatment had been legally identified. Specifically, we obtained information from social services records about youths’ placement histories following their removal from parental custody and placement into foster care. Such experiences are relevant to youth well-being (Rubin et al., 2007) and may have implications for both their evaluations of subjective social status and risk for suicidal ideation. According to social services records, 55% of youth were in the first placement of their current case (although some may have experienced prior cases and/or removal from home), while the remainder of youth (45%) had experienced multiple placement changes during their current case (*M* = 2.94, *SD* = 3.93; range: 1–29). As might be expected, the data were positively skewed (skewness statistic = 3.49, *p* < 0.001). Given the non-normal distribution, the substantial portion of youth who were in their first placement, and the potential for these youth to differ in both perceptions and mental health from youth who had repeatedly changed placements (e.g., see Koh et al., 2014; Rubin et al., 2004), placement status was recoded dichotomously to reflect whether youth were (1) versus were not (0) in their first placement.^2^

Youth were eligible to participate if they had resided at the facility for at least three days, had no observable cognitive disability (i.e., were able to adequately understand and answer our questions, were not identified by staff as having a disability), and could communicate verbally in English (because children were school-age, most spoke English in school and were completely fluent and comfortable speaking English). Because the youth were no longer in parental custody, the Presiding Judge of Juvenile Court in the county where data were collected granted permission for eligible youth to be approached and invited to participate. Further, on each day of data collection, staff familiar to the youth confirmed their interest, availability, and appropriateness to be interviewed before youth were approached. Youth provided written assent.

### Procedure and Measures

The University’s Institutional Review Board, Social Services, and the Presiding Judge of Juvenile Court approved of all study procedures. After assent was secured, youth completed a series of questionnaires during an in-person session lasting approximately one hour. Interviews were conducted in a quiet, semi-private location by a trained research assistant. Measures relevant to the present study are described here.

#### Demographic Information

At the beginning of each interview, youth provided demographic information, including their age, gender, ethnicity, race, and grade in school.

#### Subjective Social Status

Perceived social status was assessed with an adapted version of the MacArthur Scale of Subjective Social Status (Goodman et al., 2001). Youth were shown an image of a ladder with ten rungs and told to “*Think about you and other kids your age, like kids in your neighborhood or school. At the top are kids who are considered the best. At the bottom are the kids who are the lowest, in terms of their standing or how they are doing. Think about where you would fit in using this picture.”* Youth were then instructed to indicate which rung on the ladder best represented where they stood relative to other youth, with the lowest rung (1) representing the lowest standing and the highest rung (10) representing the highest standing (*M* = 6.28, *SD* = 2.51; range 1–10).

#### Suicidal Ideation and Trauma Symptomatology

Youth completed an abbreviated version of the Trauma Symptoms Checklist for Children (TSCC; Briere, 1996; Wherry et al., 2016), where they rated the frequency (ranging from 0 = *never* to 3 = *almost all the time*) with which they experienced various trauma-related symptoms across five clinical categories: anxiety (e.g., “Feeling nervous or jumpy inside”), depression (e.g., “Feeling sad or unhappy”), anger (e.g., “Getting mad and can’t calm down”), post-traumatic stress (e.g., “Can’t stop thinking about something bad that happened to me”), and dissociation (e.g., “Feeling like things aren’t real”). Of main interest in the present investigation was youths’ responses to an item on the depression subscale pertaining to suicidal ideation. This item asked about how often youth have felt like wanting to kill themselves. Scores on this item were positively skewed (*M* = 0.25, *SD* = 0.62, skewness statistic = 2.67, *p* < 0.001), with a majority (65%) of youth who endorsed suicidal ideation reporting that they only sometimes experienced such ideation (as opposed to a lot of the time or almost all of the time). Given generally low variability in the frequency with which youth experienced suicidal ideation and our broader interest in identifying youth at risk for any level of suicidal ideation, we dichotomized suicidal ideation into a yes/no index reflecting whether youth had ever felt suicidal (ranging from sometimes to almost all the time). Based on this index, 49 youth were classified as having experienced some level of suicidal ideation. A total trauma symptomatology score was also computed to account for youths’ general level of trauma symptoms, including depression, in main analyses. All items except for the aforementioned suicide item were included in this total trauma symptomatology index. Cronbach’s alpha = 0.89 was comparable to that reported in other studies with maltreated samples (e.g., Wherry & Dunlop, 2018).

#### General Cognitive Functioning

Youths’ overall cognitive functioning was also assessed, given that this area of functioning has been found in prior work to be relevant to self-perceptions and mental health symptoms, including suicidal ideation (Bredemeier & Miller, 2015; Nock & Kazdin, 2002). Youth completed the Digit Span task, a widely-used measure of working memory for children ages 6–16 (WISC-IV; Wechsler, 2003) that includes simple (Forward) and complex (Backward) tasks. Only the simple task was administered in the present investigation, given evidence that both tasks load on the same factor (e.g., Colom et al., 2005; Engle et al., 1999). Youth were read a string of digits at a rate of approximately one digit per second and were asked to repeat the string verbatim. String length progressively increased until youth failed two presentations of the same length within a trial. Age-standardized scores were computed for the total number of digit strings correctly recalled, with higher scores reflecting greater working memory capacity relative to youth age.

#### Maltreatment History

Social service records for each youth detailed their current and prior child welfare case histories. Each of these records was reviewed by trained research assistants or doctoral students and coded according to the Maltreatment Classification System (MCS; Barnett et al., 1993), a well-validated and reliable method of classifying child maltreatment experiences (English et al., 2005). The MCS utilizes all available information from social services records rather than relying solely on official designations or case dispositions, and thus produces independent determinations of maltreatment. The MCS contains operational criteria to code all of the major subtypes of maltreatment, including neglect (i.e., failure to provide for children’s basic physical needs or supervision), physical abuse (i.e., intentional infliction of physical injury), sexual abuse (i.e., attempted or actual sexual contact, including exposure to pornography or adult sexual activity) and emotional maltreatment (i.e., extreme thwarting of emotional needs, e.g., for psychological safety, acceptance, self-esteem, and age-appropriate autonomy; note, this form of maltreatment was not coded in the present investigation, given a general lack of documentation in social service records). Before independently coding cases based on these criteria, coders first established acceptable reliability on a subset of cases (*K*s ranged from 0.83–0.90). In addition, coders continued to double-code cases throughout the study to ensure that reliability of coding remained high (*K*s > 0.80).

### Analytic Plan

Analyses proceeded in three steps. First, we conducted descriptive analyses to characterize rates of suicidal ideation and subjective social status in our maltreated sample. These included correlations, chi-squared tests, and independent samples *t*-tests, as appropriate. Second, we tested main study hypotheses concerning the links between child maltreatment (namely abuse versus neglect), subjective social status, and suicidal ideation via a hierarchical logistic regression that controlled for other individual and experiential characteristics (e.g., gender, trauma symptoms) known to be associated with suicidal ideation in maltreated youth. Variables entered at Step 1 included gender, abuse, Latinx ethnicity, developmental status, placement history, concurrent trauma symptoms, cognitive functioning, and subjective social status. At Step 2, we entered the interaction between abuse and subjective social status, followed by the interaction between developmental status and subjective social status at Step 3. Significant interactions were probed using simple slopes analysis, and nested models were compared via likelihood-ratio tests. Third, we conducted an exploratory logistic regression analysis testing whether the associations among suicidal ideation, abuse, and subjective social status varied based on developmental status. Steps 1–3 of the model were identical to that of our main analyses; however, we also included the two-way interaction between abuse and developmental status at Step 4 and the three-way interaction between abuse, subjective social status, and developmental status at Step 5. Nested models were again compared using likelihood-ratio tests.

## Results

### Preliminary Analyses

Descriptive statistics and correlations are presented in Table 1. Approximately 18% of the sample reported a history of suicidal ideation. At the bivariate level, rates did not significantly vary based on developmental status (adolescent v. preadolescent) or Latinx ethnicity, *χ*^*2*^s (1) ≤ 0.90, *ps* ≥ 0.34, but did vary by gender, with girls reporting proportionally greater suicidal ideation than boys (22% v. 13%, respectively), χ^*2*^(1) = 4.23, *p* = 0.04. Marginal effects of abuse, χ^*2*^(1) = 3.09, *p* = 0.08, were also evident, such that proportionally more abused than neglected youth reported suicidal ideation (22% v. 14%, respectively).

**Table 1:** Descriptive Statistics and Bivariate Correlations Between Main Study Variables (*N* = 279)

Variables	*M*	*SD*	1	2	3	4	5	6	7	8	9
1. Female	0.52		-								
2. Abuse	0.49		0.07	-							
3. Latinx Ethnicity	0.53		-0.06	-0.06	-						
4. Developmental Status	0.37		-0.01	0.28***	-0.15*	-					
5. Placement History	0.55		0.09	-0.08	0.004	-0.13*	-				
6. Trauma Symptoms	0.94	0.50	0.16**	0.05	-0.08	-0.02	0.04	-			
7. Digit Span	7.37	2.05	0.08	0.18**	-0.23***	0.26***	-0.05	-0.03	-		
8. Subjective Social Status	6.29	2.50	-0.12*	-0.12*	0.13*	-0.22*	0.01	-0.20**	-0.13*	-	
9. Suicidal Ideation	0.18		0.12*	0.10^t^	0.02	0.06	0.02	0.50***	-0.001	-0.26***	-

Reference groups for the first five dichotomous demographic variables are as follows: male gender, neglect exposure, non-Latinx ethnicity, preadolescents, and multiple placement changes. Suicidal ideation was dichotomized (1 = yes, 0 = no). Point-biserial correlations and Phi coefficients are included, where appropriate

^*^p < 0.05, **p < 0.01, ***p < 0.001, ^t^ *p* = 0.08

With regard to subjective social status, ratings differed based on developmental status, Latinx ethnicity, gender, and abuse history, *t*s (277) ≥|2.03|, *p*s ≤ 0.04, *d*s ≥|.24|. Adolescents reported significantly lower subjective social status (*M* = 5.56, *SD* = 2.07) than preadolescents (*M* = 6.79, *SD* = 2.64); non-Latinx youth reported significantly lower subjective social status (*M* = 6.08, *SD* = 2.59) than Latinx youth (*M* = 6.53, *SD* = 2.36); girls reported significantly lower subjective status (*M* = 6.00, *SD* = 2.41) than boys (*M* = 6.61, *SD* = 2.57); and abused youth (*M* = 5.98, *SD* = 2.49) reported significantly lower subjective social status than neglected youth (*M* = 6.59, *SD* = 2.48).

Correlations among main study variables showed that both suicidal ideation and subjective social status were significantly associated with concurrent trauma symptoms per the TSCC: Youth who endorsed suicidal ideation tended to report higher levels of concurrent trauma symptoms (*r* = 0.47, *p* < 0.001), and higher levels of such symptoms were associated with lower subjective social status (*r* = -0.23, *p* < 0.001). Follow-up examination of the individual subscales of the TSCC showed that each subscale was positively associated with suicidal ideation, with correlations ranging from *r* = 0.23 (dissociation) to *r* = 0.59 (depression), *p*s < 0.001. Similarly, all subscales except for disassociation were negatively associated with subjective social status, with correlations ranging from *r* = -0.13 (anxiety) to *r* = -0.24 (depression and post-traumatic stress), *p*s < 0.05. Cognitive functioning per the Digit Span was negatively associated with subjective social status, *r* = -0.13, *p* < 0.05, but was unrelated to suicidal ideation, *p* = 0.60. Finally, with regard to developmental status, adolescents were more likely to have experienced abuse, *r* = 0.28, *p* < 0.001, and multiple changes in placement, *r* = -0.13, *p* < 0.05, than preadolescents; adolescents also showed higher cognitive functioning, *r* = 0.26, *p* < 0.001, and were somewhat less likely to be of Latinx ethnicity, *r* = -0.15, *p* < 0.001.

### Main Analyses

**Fig. 1:**
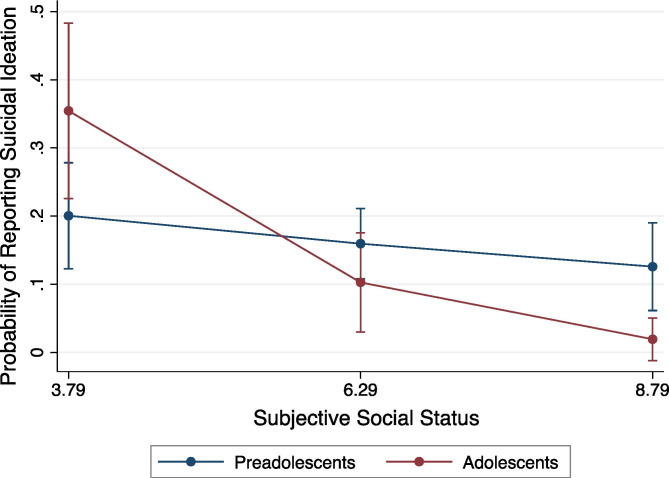
Interaction between subjective social status and developmental status in predicting risk for suicidal ideation (yes/no). Subjective social status is plotted at ± 1 SD above and below the mean and at the mean. Error bars represent 95% confidence intervals

As evident in Table 2, the model was statistically significant at all steps, as was the proportion of variance explained by Step 3, LR χ^2^ (2) = 11.52, *p* = 0.003. Concurrent trauma symptoms (Odds ratio [OR] = 23.36, 95% CI [8.97, 60.85]) and developmental status (OR = 51.69, 95% CI [4.20, 636.06]) were positively related to suicidal ideation in youth. However, the latter association was subsumed by a significant interaction with subjective social status (OR = 0.47, 95% CI [0.29, 0.77]), generally consistent with study hypotheses. Simple slopes analyses, shown in Fig. 1, revealed that at low levels of subjective social status (-1SD), adolescents reported a significantly *higher* probability of suicidal ideation than preadolescents, *b* = 0.14, SE = 0.07, *p* = 0.05, 95% CI [0.00, 0.31]. The opposite trend emerged at high levels of subjective social status (+ 1SD), with adolescents reporting a significantly *lower* probability of suicidal ideation relative to preadolescents, *b* = -0.12, SE = 0.04, *p* = 0.001, 95% CI [-0.19, -0.05] (there were no statistically significant group differences at the mean of subjective social status). The overall slope for preadolescents was nonsignificant, suggesting that subjective social status was not related to whether or not they endorsed suicidal ideation, at least after accounting for other variables in the model (e.g., trauma symptoms), *b* = -0.01, *SE* = 0.01, *p* = 0.44, 95% CI [-0.02, 0.01]. Contrary to our other predictions and preliminary bivariate analyses, neither abuse nor the interaction between abuse and subjective social status was significantly related to suicidal ideation in youth.

**Table 2:** Logistic Regression Analysis Predicting Suicidal Ideation in Maltreated Adolescents and Preadolescents (*N* = 279)

Model	B	SE	Wald χ^2^(1)	Odds Ratio (OR)	95% Confidence Interval (OR)
**Step 1**					
Female	0.05	0.40	0.13	1.05	[0.48, 2.31]
Abuse	0.39	0.42	0.92	1.48	[0.64, 3.41]
Latinx Ethnicity	0.66	0.41	1.59	1.94	[0.86, 4.41]
Developmental Status	0.23	0.45	0.51	1.26	[0.52, 3.01]
Placement History	0.20	0.39	0.51	1.23	[0.56, 2.68]
Concurrent Emotional Symptoms	2.96	0.46	6.39***	19.25	[7.77, 47.73]
Digit Span	-0.08	0.11	-0.69	0.93	[0.75, 1.15]
Subjective Social Status	-0.24	0.08	-2.97**	0.78	[0.67, 0.92]
Overall Model	LR χ^2^ (8) = 84.64, *p* < 0.001				
**Step 2**					
Female	0.06	0.40	0.15	1.06	[0.48, 2.34]
Abuse	0.67	0.98	0.68	1.96	[0.28, 13.59]
Latinx Ethnicity	0.67	0.42	1.59	1.95	[0.86, 4.44]
Developmental Status	0.21	0.45	0.46	1.23	[0.51, 2.99]
Placement History	0.21	0.39	0.52	1.23	[0.56, 2.69]
Concurrent Emotional Symptoms	2.96	0.46	6.37***	19.29	[7.76, 47.95]
Digit Span	-0.08	0.11	-0.72	0.92	[0.75, 1.15]
Subjective Social Status	-0.22	0.11	-1.96*	0.80	[0.65, 0.99]
Abuse X Subjective Social Status	-0.05	0.15	-0.32	0.95	[0.69, 1.30]
Overall Model	LR χ^2^ (9) = 84.74, *p* < 0.001				
**Step 3**					
Female	0.27	0.42	0.63	1.31	[0.57, 2.99]
Abuse	-0.45	1.06	-0.43	0.64	[0.08, 5.04]
Latinx Ethnicity	0.73	0.44	1.66	2.08	[0.88, 4.93]
Developmental Status	3.94	1.28	3.08**	51.69	[4.20, 636.06]
Placement History	0.18	0.41	0.43	1.20	[0.53, 2.69]
Concurrent Emotional Symptoms	3.15	0.49	6.45***	23.37	[8.97, 60.85]
Digit Span	-0.04	0.11	-0.34	0.96	[0.77, 1.20]
Subjective Social Status	-0.14	0.11	-1.21	0.87	[0.70, 1.08]
Abuse X Subjective Social Status	0.11	0.16	0.70	1.12	[0.81, 1.55]
Development X Subjective Social Status	-0.75	0.24	-3.04**	0.47	[0.29, 0.77]
Overall Model	LR χ^2^ (10) = 96.16, *p* < 0.001,				

*Note*. The change in model fit between Steps 1 and 3 and Steps 2 and 3 was significant, p < .01. Reference groups for the first five dichotomous demographic variables are as follows: male gender, neglect exposure, non-Latinx ethnicity, preadolescents, and multiple placement changes

^*^p < 0.05, **p < 0.01, ***p < 0.001

### Exploratory Analyses

Although we did not find evidence that abuse was related to suicidal ideation directly or in conjunction with subjective social status, it was possible that these associations varied based on youths’ developmental status. Thus, we reconducted analyses above with the addition of the two-way interaction between abuse and developmental status and the three-way interaction among abuse, subjective social status, and developmental status. The overall model was statistically significant, LR χ^2^ (12) = 98.92, *p* < 0.001, however the proportion of variance explained by Steps 4 and 5 was non-significant, *p* ≥ 0.11 (see Table 3). Thus, abuse type did not appear to be related to suicidal ideation among the youth in our sample, including in combination with subjective social status and developmental status.

**Table 3:** Exploratory Logistic Regression Analysis Predicting Suicidal Ideation in Maltreated Adolescents and Preadolescents (*N* = 279)

Model	*b*	SE	Wald χ^2^ (1)	Odds Ratio (OR)	95% Confidence Interval (OR)
**Step 1**					
Female	0.05	0.4	0.13	1.05	[0.48, 2.31]
Abuse	0.39	0.42	0.92	1.48	[0.64, 3.41]
Latinx Ethnicity	0.66	0.41	1.59	1.94	[0.86, 4.41]
Developmental Status	0.23	0.45	0.51	1.26	[0.52, 3.01]
Placement History	0.2	0.39	0.51	1.23	[0.56, 2.68]
Trauma Symptoms	2.96	0.46	6.39***	19.25	[7.77, 47.73]
Digit Span	-0.08	0.11	-0.69	0.93	[0.75, 1.15]
Subjective Social Status	-0.24	0.08	-2.97**	0.78	[0.67, 0.92]
Overall Model	LR χ^2^ (8) = 84.64, *p* < 0.001				
**Step 2**					
Female	0.06	0.40	0.15	1.06	[0.48, 2.34]
Abuse	0.67	0.98	0.68	1.96	[0.28, 13.59]
Latinx Ethnicity	0.67	0.42	1.59	1.95	[0.86, 4.44]
Developmental Status	0.21	0.45	0.46	1.23	[0.51, 2.99]
Placement History	0.21	0.39	0.52	1.23	[0.56, 2.69]
Trauma Symptoms	2.96	0.46	6.37***	19.29	[7.76, 47.95]
Digit Span	-0.08	0.11	-0.72	0.92	[0.75, 1.15]
Subjective Social Status	-0.22	0.11	-1.96*	0.80	[0.65, 0.99]
Abuse X Subjective Social Status	-0.05	0.16	-0.32	0.95	[0.69, 1.30]
Overall Model	LR χ^2^ (9) = 84.74, *p* < 0.001				
**Step 3**					
Female	0.27	0.42	0.63	1.31	[0.57, 2.99]
Abuse	-0.45	1.06	-0.43	0.64	[0.08, 5.04]
Latinx Ethnicity	0.73	0.44	1.66	2.08	[0.88, 4.93]
Developmental Status	3.94	1.28	3.08**	51.69	[4.20, 636.06]
Placement History	0.18	0.41	0.43	1.20	[0.53, 2.69]
Trauma Symptoms	3.15	0.49	6.45***	23.37	[8.97, 60.85]
Digit Span	-0.04	0.11	-0.34	0.96	[0.77, 1.20]
Subjective Social Status	-0.14	0.11	-1.21	0.87	[0.70, 1.08]
Abuse X Subjective Social Status	0.11	0.16	0.70	1.12	[0.81, 1.55]
Development X Subjective Social Status	-0.75	0.24	-3.04**	0.47	[0.29, 0.77]
Overall Model	LR χ^2^ (10) = 96.16, *p* < 0.001				
**Step 4**					
Female	0.27	0.42	0.63	1.30	[0.57, 3.00]
Abuse	-0.71	1.18	-0.60	0.49	[0.05, 4.96]
Latinx Ethnicity	0.76	0.44	1.71	2.14	[0.89, 5.12]
Developmental Status	3.62	1.42	2.54*	37.53	[2.30, 612.23]
Placement History	0.19	0.41	0.45	1.20	[0.53, 2.71]
Trauma Symptoms	3.19	0.50	6.38***	24.32	[9.13, 64.80]
Digit Span	-0.04	0.11	-0.34	0.96	[0.77, 1.20]
Subjective Social Status	-0.14	0.11	-1.27	0.88	[0.70, 1.08]
Abuse X Subjective Social Status	0.14	0.17	0.80	1.14	[0.82, 1.59]
Development X Subjective Social Status	-0.75	0.25	-3.06**	0.47	[0.29, 0.76]
Abuse X Development	0.52	1.05	0.49	1.68	[0.21, 13.12]
Overall Model	LR χ^2^ (11) = 96.40, *p* < 0.001				
**Step 5**					
Female	0.26	0.43	0.62	1.30	[0.56, 3.03]
Abuse	-0.19	1.27	-0.15	0.83	[0.07, 9.91]
Latinx Ethnicity	0.64	0.45	1.43	1.90	[0.79, 4.60]
Developmental Status	7.56	3.41	2.21*	1924.53	[2.38, 1551622.00]
Placement History	0.13	0.42	0.30	1.13	[0.50, 2.58]
Trauma Symptoms	3.32	0.52	6.39***	27.57	[9.97, 76.21]
Digit Span	-0.03	0.11	-0.23	0.97	[0.78, 1.22]
Subjective Social Status	-0.10	0.11	-0.85	0.91	[0.72, 1.14]
Abuse X Subjective Social Status	0.05	0.18	0.79	1.05	[0.73, 1.51]
Development X Subjective Social Status	-1.57	0.70	-2.27*	0.21	[0.05, 0.81]
Abuse X Development	-4.40	3.68	-1.20	0.01	[0.00, 16.54]
Abuse X Subjective Social Status X Development	1.02	0.73	1.40	2.77	[0.66, 11.61]
Overall Model	LR χ^2^ (12) = 98.92, *p* < 0.001				

The change in fit between Steps 3 and 4 and Steps 4 and 5 was non-significant, *p* ≥ 0.11. Reference groups for the first five dichotomous demographic variables are as follows: male gender, neglect exposure, non-Latinx ethnicity, preadolescents, and multiple placement changes

^*^p < 0.05, **p < 0.01, ***p < 0.001

## Discussion

Suicide is a leading cause of death among young people, including adolescents, globally, with recent decades seeing a rising trend in suicide rates among youth ages 10–24 years (Curtin & Heron, 2019). Although prior work implicates child maltreatment as a potent risk factor for suicidality in youth, knowledge of factors linking child maltreatment and suicidal ideation remains limited. In the present study, we examined how maltreated youths’ own perceptions of their social status related to adolescents’ and pre-adolescents’ risk for suicidal ideation. Our approach was fairly simple, but represents an important first step in understanding how subjective perceptions relate to suicidal ideation in a population that has not been featured prominently in research on subjective social status and suicidal ideation, despite heightened risk for negative self-perceptions and suicidality. Consistent with work with other adolescent populations, our findings reveal that subjective social status is relevant to risk for suicidal ideation in maltreated adolescents and thus may provide potential directions for intervention efforts aimed at reducing suicidality in this population.

Our findings suggest that perceptions of social status operate differently between adolescent- and preadolescent-aged maltreated youth. Among adolescents, subjective social status was associated with lower or higher risk for suicidal ideation, depending on how the adolescents’ perceived their place in their social hierarchy. Adolescents who viewed themselves as lower in social standing reported significantly greater suicidal ideation, whereas adolescents who viewed themselves as higher in social standing reported significantly lower suicidal ideation. These associations align with recent empirical and theoretical work emphasizing the salience of status-related perceptions and social status concerns during adolescence, including in relation to mental health symptoms like suicidal ideation (Goodman et al., 2001; Ko et al., 2014; Odgers, 2015; Steinberg & Morris, 2001). The lack of a unique association between these perceptions and risk for suicidal ideation in preadolescents (at least after accounting for other aspects of youths’ functioning) may indicate that status-related perceptions matter less in relation to suicidal ideation during preadolescence. Alternatively, or in addition, perhaps these perceptions are less accurate or sophisticated at younger ages and thus are not strongly associated with behavioral tendencies. In potential support of the latter possibility, we found developmental differences in perceptions of social status: Preadolescents reported more optimistic views overall of their social standing than did adolescents, despite general similarities in experiences (e.g., exposure to maltreatment, removal from home). Nonetheless, given that these perceptions have not been studied extensively in maltreated samples, additional work is needed to understand the development of these perceptions, how they track objective indicators of social status and experience, and their malleability in maltreated youth. This knowledge may inform efforts to prevent youth suicide and improve broader functioning in a high-risk population.

Our results also support theoretical models emphasizing the connections between negative self-evaluations and suicidal ideation in adolescence. Cognitive behavioral models of adolescent suicide contend that cognitive distortions (e.g., negative cognitive triad, black-and-white thinking, overgeneralization, hopelessness) play a causal role in suicidal thoughts and behavior (Spirito et al., 2011). Though such distortions are prevalent among maltreated youth (Ponce et al., 2004; Toth et al., 1997, 2000), research focused on their associations with suicidal ideation in maltreated populations has been lacking (for a critical examination of the literature, see Miller et al., 2013). Findings from the present study, combined with those of limited prior work (Kim & Ko, 2020; Ko et al., 2014; Miller & Esposito-Smythers, 2013), suggest that adolescents’ perceptions of themselves, their lives, and future—including perceptions of their social standing compared to their own self-defined peers— may be a promising line of research in understanding and preventing risk for suicidality specifically in maltreated youth. Of importance, such perceptions are easy to assess and do not require objective markers of social status or SES, as they reflect the youths' own perceptions based on their self-defined peers. Thus, they may be more modifiable than other risk factors linked to maltreated youths’ environment and may represent a feasible target for efforts aimed at reducing risk. However, given that perceptions were unrelated to *preadolescents*’ endorsement of suicidal ideation, understanding other sets of cognitions and experiences relevant to risk for suicidality in young maltreated youth will be a much-needed addition to extant work.

In contrast to prior maltreatment research (e.g., Dunn et al., 2013; Duprey et al., 2020; Thompson et al., 2005), our results did not reveal that aspects of the maltreatment or foster care experience were related to suicidal ideation in either preadolescents or adolescents. We also did not find that maltreatment, namely abuse versus neglect, interacted with subjective social status or developmental status, at least in our sample, to predict suicidal ideation. The co-occurrence of maltreatment subtypes and other forms of adversity (e.g., poverty, social service involvement, violence exposure) in our sample may have obscured potential group differences in effects. Had we been able to contrast the youths’ reports with those of a demographically matched comparison sample (who, due to low base rates of suicidal ideation, were unable to be included in the present study) or incorporate data regarding the severity, chronicity, and developmental timing of the specific types of maltreatment experienced, different findings may have emerged. Recent conceptual work also points to the need to assess other factors (e.g., features of the environment and social context or individual differences factors) beyond the specific adverse events (e.g., abuse, neglect) to which children are exposed that may also determine their responses to such adversity (Smith & Pollak, 2020). This will be an important direction for future work, as will be work that assesses, with greater precision, how different aspects of the foster care experience (e.g., placement type, trajectories of placement stability) relate to suicidal ideation in maltreated youth, given the potential impacts of these experiences on youth well-being (e.g., Anderson, 2011; Rubin et al., 2008).

The present study makes a novel contribution in evaluating whether perceived social status relates to risk for suicidal ideation in maltreated youth; however, limitations must be noted. First, our findings cannot speak to directionality, that is, whether subjective social status *causes* suicidal ideation or is caused *by* suicidal ideation. Rigorous longitudinal and experimental studies are needed to disentangle causality but also to test whether status-related perceptions can be experimentally altered in ways that lower risk for suicidal ideation. Regardless, our results offer valuable information for identifying adolescents at risk for suicidal ideation, suggesting that those who might be at risk for such behavior, and hence most in need of intervention, are those who view themselves as lower on the social ladder. Second, youth in the present study came from low-income backgrounds. Although variation existed in youths’ subjective social status, future work should include a broader range of youth to separate the unique effects of poverty, social class, and maltreatment on perceptions and their associations with suicidal ideation. Third, we only assessed one set of self-evaluations in maltreated youth, doing so via a single (albeit widely used) item. Understanding how maltreated youths’ subjective social status tracks other self-evaluations and youths’ broader views of the world will be of interest in future work. Viewing themselves as lower ranked in society may be one “symptom” of a larger constellation of negative self-perceptions maltreated youth hold about themselves. Further, how maltreated youth view the world (safe/unsafe, predictable/unpredictable) as a result of their experiences may well shape not only their perceptions of their social standing relative to others, but the impact of these perceptions on mental health. Finally, as youth are increasingly being exposed to inequality across a number of domains (e.g., socioeconomic, racial, educational; Duncan & Murnane, 2011; Odgers & Adler, 2018), research will also need to consider more complex and multi-dimensional approaches to measuring subjective social status than that used in the present study. Understanding who youth are comparing themselves to and in what context will provide a more nuanced picture of how youth perceive themselves relative to others and the role that these perceptions play in suicidal ideation and other mental health symptoms.

In summary, the present findings suggest that perceptions of social status may hold unique relevance to maltreated adolescents’ risk for suicidal ideation. When adolescents view themselves as lower ranked in society, they may be at heightened risk for suicidal ideation, whereas viewing themselves as higher ranked in society may be protective in this regard. Future work is needed to test the generalizability of these associations to broader groups of youth but is especially important given staggering increases in youth suicide rates and growing inequality in the lives of children and adolescents globally. An important next step in such work will be to examine when in development status-related perceptions are first internalized and the effects of these perceptions on suicidal ideation and other aspects of mental health in diverse populations and across development. This would offer much-needed insight into potential methods of promoting child and adolescent well-being.

## Footnotes

1 Demographically-matched community youth (*n* = 114) were also interviewed; however, their base rates of suicidal ideation (8%; much lower than that reported by the maltreated youth) were too low to include these youth in analyses.

2 All reported analyses were also conducted with placement history scored continuously (log-transformed). Because patterns were highly similar across models, only models including the dichotomous placement index are presented.
